# Supplementary material for: Content-rich biological network constructed by mining PubMed abstracts
Source: BMC Bioinformatics. 2004 Oct 8;5:147. doi: 10.1186/1471-2105-5-147 (PMC528731; doi:10.1186/1471-2105-5-147)
Supplement: Additional File 2 — The original results of the above study (non-essential files are deleted to keep the file size under the limit set by BMC bioinformatics). [file 1471-2105-5-147-S2.bz2 › chilibotAdditionalFile2/dip10/260ID8816472E1029/html/BYR2_STE4.html]

 


 **BYR2** and **STE4** 
  
Found 7 abstracts in PubMed, retrieved 7.  
 

 What does Google say? 
 PDF only 
| .edu only 

---

**Interactive relationship** (e.g. stimulation, inhibition, etc)

**Neutral relationship**- Both  **Ste4**  and Ras1 act upstream of  **Byr2**  and act at least partially independently.  Ref: 8816472 Mol Cell Biol, 1996
- **Ste4**  binds to the regulatory region of  **Byr2** .  Ref: 8816472 Mol Cell Biol, 1996
- Here, we show that the individual SAM domains of  **Ste4**  and  **Byr2**  are monomeric at low concentrations and bind to each other in a 1 1 stoichiometry with a relatively weak dissociation constant of 56 3 microm.  Ref: 12171939 J Biol Chem, 2002
- **Ste4**  and Ras1 interact with the regulatory domain of  **Byr2**  directly.  Ref: 9315645 Mol Cell Biol, 1997
- **Byr2**  is a mitogen activated protein kinase kinase kinase that is regulated by  **Ste4** .  Ref: 12171939 J Biol Chem, 2002
- We find that the  **Ste4**  LZ domain is trimeric and, when included with the  **Ste4**  SAM domain, yields a 3 1  **Ste4**  LZ SAM  **Byr2**  SAM complex with a tight dissociation constant of 19 4 nm.  Ref: 12171939 J Biol Chem, 2002
- These results suggest that the  **Ste4**  LZ SAM protein may recognize multiple binding sites on  **Byr2**  SAM, indicating a new mode of oligomeric organization for SAM domains.  Ref: 12171939 J Biol Chem, 2002
- A screen for S. pombe proteins that interact with  **Byr2**  in a two hybrid system led to the isolation of  **Ste4** , a protein that is known to be required for sexual function.  Ref: 8816472 Mol Cell Biol, 1996
- The regulatory region can be divided into a sterile alpha motif SAM that binds  **Ste4** , a Ras1 binding domain RBD that binds activated Ras1, and a catalytic binding domain CBD that interacts with the  **Byr2**  kinase domain.  Ref: 9810469 Biochimie, 1998

**Non-interactive relationship** (e.g. studied together, co-existance, homology, etc.)

- **Byr2**  and  **Ste4**  are two SAM domain containing proteins in the mating pheromone response pathway of the fission yeast, Schizosaccharomyces pombe.  Ref: 12171939 J Biol Chem, 2002
- Single copies of an approximately 65 70 residue domain are shown to be present in the sequences of 14 eukaryotic proteins, including yeast  **byr2** , STE11,  **ste4** , and STE50, which are essential participants in sexual differentiation.  Ref: 8528090 Protein Sci, 1995
- Here, we define the domains of  **Byr2**  that bind  **Ste4**  and Ras1 and show that the  **Byr2**  regulatory domain binds to the catalytic domain in the two hybrid system.  Ref: 9315645 Mol Cell Biol, 1997
- **Byr2**  functions downstream of  **Ste4** , Ras1, and the membrane associated receptor coupled heterotrimeric G protein alpha subunit, Gpa1.  Ref: 9315645 Mol Cell Biol, 1997
- 1 876 5887 showed that the isolated SAM domain of  **Byr2**  binds a fragment of  **Ste4**  that contains both a leucine zipper  **Ste4**  LZ domain as well as a SAM domain, suggesting that  **Byr2**  SAM and  **Ste4**  SAM may form a hetero oligomer.  Ref: 12171939 J Biol Chem, 2002
- Oligomerization dependent association of the SAM domains from Schizosaccharomyces pombe  **Byr2**  and  **Ste4** .  Ref: 12171939 J Biol Chem, 2002
- Deletion of rad24 or rad25 increased sporulation efficiency in ras1 null diploid cells but not in  **byr2** ,  **ste4** , byr1, and spk1 null cells.  Ref: 12242289 Mol Cell Biol, 2002
- Identification of  **Ste4**  as a potential regulator of  **Byr2**  in the sexual response pathway of Schizosaccharomyces pombe.  Ref: 8816472 Mol Cell Biol, 1996
